# Comparison of the Outcomes of the Proximal Femur Plate (PFP) and Proximal Femoral Nail (PFN) in a Patient Presenting With Trochanteric Femur Fracture

**DOI:** 10.7759/cureus.93745

**Published:** 2025-10-02

**Authors:** Muhammad Mannan, Mohammad Osama, Basit Mukhtar, Nayan Shrivastava, Faisal Karim, Zawar Ahmad, Rehan Raza Shan, Muhammad Zeeshan Akram, Muhammad Tayyab

**Affiliations:** 1 Trauma and Orthopaedics, Ghurki Trust and Teaching Hospital, Lahore, PAK; 2 Trauma and Orthopaedics, Sheikh Zayed Medical College and Hospital, Rahim Yar Khan, PAK; 3 Orthopaedic Surgery, University Hospitals Birmingham NHS Foundation Trust, Birmingham, GBR; 4 Trauma and Orthopaedics, Pakistan Institute of Medical Sciences, Islamabad, PAK; 5 Trauma and Orthopaedics, Queen Elizabeth Hospital Birmingham, Birmingham, GBR; 6 Orthopaedics, Sancheti Institute for Orthopedics and Rehabilitation, Pune, IND; 7 Trauma and Orthopaedics, University Hospitals Birmingham NHS Foundation Trust, Birmingham, GBR; 8 Trauma and Orthopaedics, Kettering General Hospital, Kettering, GBR; 9 Trauma and Orthopaedics, University Hospitals Birmingham NHS Foundation Trust, Heartlands Hospital, Birmingham, GBR; 10 Trauma and Orthopaedics, Bradford Teaching Hospitals NHS Foundation Trust, Bradford, GBR

**Keywords:** fracture healing, proximal femoral nailing (pfn), proximal femur plate (pfp), surgical site infection, trochanteric fracture

## Abstract

Objective: This study aimed to compare the outcomes of proximal femoral nailing (PFN) and proximal femur plate (PFP) in patients with trochanteric fractures. The primary objective was to assess fracture union time, while secondary objectives included operative duration, surgical site infection (SSI), and length of hospital stay.

Study design: This is a prospective comparative study.

Setting and duration: The study was conducted at the Department of Orthopaedics, Pakistan Institute of Medical Sciences (PIMS), Islamabad, from September 21, 2023, to September 20, 2024, following approval of the study synopsis by the College of Physicians and Surgeons Pakistan (CPSP).

Materials and methods: A total of 60 patients who met the inclusion criteria and underwent surgical treatment for trochanteric fractures were enrolled. Patients were allocated to one of two groups based on the surgical procedure performed: Group A underwent PFN, and Group B underwent PFP. No randomization was performed; patients were assigned based on the procedure received. All fractures were classified according to the AO Foundation and Orthopaedic - Trauma Association (AO/OTA) 31-A system and categorized as stable or unstable. Postoperative outcomes were assessed at one, three, and four months, focusing on SSI, operative time, and fracture healing.

Results: In the PFN group, patient ages ranged from 50 to 80 years (median: 60 years), while in the PFP group, the median age was 58 years. SSI occurred in two patients (6.7%) in the PFN group and in one patient (3.3%) in the PFP group (p = 0.500). The mean operative duration was significantly longer in the PFN group (79.10 ± 12.78 minutes) compared to the PFP group (64.13 ± 12.92 minutes) (p < 0.0001). Similarly, fracture healing time was longer in the PFN group (12.57 ± 1.79 weeks) compared to the PFP group (10.60 ± 1.10 weeks) (p < 0.0001). Stratified analyses confirmed that these differences were most evident in stable AO/OTA 31-A1 and selected A2 patterns.

Conclusion: While SSI rates were similar between the groups, PFP demonstrated shorter operative time and earlier fracture union compared to PFN. These findings suggest that PFP may be considered in selected stable fracture patterns, particularly in resource-constrained settings, whereas PFN remains the preferred option for unstable fractures.

## Introduction

Trochanteric fractures occur in the proximal femur, with extension to the medullary canal. It involves the area from the extracapsular basilar neck to the lesser trochanter [1]. In the elderly population, these fractures are very common [2]. The incidence of trochanteric fractures has been increasing. This is due to greater life expectancy, which leads to more prevalent osteoporosis. According to recent studies, an estimated 4.5-6.26 million hip fractures will occur worldwide by 2050, with 50% of cases predicted in the Asian subcontinent. These fractures may be unstable, which are characterized by features such as extension into the subtrochanteric region, lateral wall blowout, comminuted posteromedial walls, reverse oblique patterns, and reverse oblique fracture variants [2,3].

The frequency of intertrochanteric femur fractures increases with age. They are found to be three times more prevalent in males than in females. Most fractures result from osteoporosis and are caused by minor trauma in elderly individuals [4]. Depending on the patient's condition and urgency of treatment, management can be either conservative or surgical [5]. Conservative management, once common, is now strongly discouraged due to its association with high morbidity and mortality in older patients. Surgical fixation has therefore become the mainstay of treatment.

Several internal fixation devices are used for intertrochanteric fractures. Extramedullary systems include the dynamic hip screw (DHS), compression hip screw (CHS), dynamic condylar screw (DCS), and proximal femoral locking compression plate (PFLCP), while intramedullary systems include the intramedullary hip screw (IMHS), proximal femoral nail (PFN), and proximal femoral nail antirotation (PFNA). Both approaches have gained empirical support. Extramedullary sliding screws, such as the DHS, were previously considered the gold standard, but now intramedullary devices are generally preferred [3]. Several studies have shown that intramedullary fixation for unstable fractures has better outcomes, whereas extramedullary devices result in higher complication rates and inferior functional outcomes [6,7].

Karim et al. [4] compared PFN and PFP. The study reported a mean operative time of 75.12 ± 6.13 minutes for PFN and 60.18 ± 5.12 minutes for PFP. The mean times to fracture union were 14.31 ± 3.12 weeks for PFN and 12.41 ± 0.38 weeks for PFP. There was no surgical site infections in either group. These results support the idea that both implants can be effective, despite the difference in operative duration and union time.

Importantly, fracture classification plays a crucial role in implant selection. The AO Foundation and Orthopaedic - Trauma Association (AO/OTA) 31-A system classifies fractures as A1 (stable), A2 (with comminution), and A3 (reverse oblique and unstable patterns). Stable fractures may be managed by either extramedullary or intramedullary fixation, but unstable patterns generally require intramedullary devices such as PFN [8,9].

We aimed to compare the outcomes of PFN and PFP in treatment of trochanteric fractures in our local population. The main outcomes are operating time, time to fracture union, and wound infection. With addition of AO/OTA classification and stability, we hope to provide more clinically applicable information for the surgeon to choose the right implants.

## Materials and methods

This prospective comparative study was carried out at Department of Orthopaedics, Pakistan Institute of Medical Sciences (PIMS), Islamabad, from September 21, 2023, to September 20, 2024. Synopsis of the study was also approved by the Research Department of the College of Physicians and Surgeons Pakistan (CPSP).

Sixty patients were chosen by non-probability consecutive sampling. Included patients were between 50 and 80 years of age, either sex, trochanteric femur fracture diagnosed, and of American Society of Anaesthesiologists (ASA) grade I-III. The exclusion and inclusion criteria are presented in Table 1.

**Table 1:** Inclusion and exclusion criteria ASA: American Society of Anaesthesiologists

Inclusion Criteria	Exclusion Criteria
Patients aged between 50–80 years	Type I fracture (Jensen–Michaelsen classification)
Either gender	History of osteoarthritis
Diagnosed with trochanteric femur fracture (as per operational definition)	Pathological fracture
ASA Physical Status Classification I–III	Bilateral femur fracture
Underwent surgical management for trochanteric fracture	

The sample size was estimated using the OpenEpi web calculator, with 80% power and 5% significance level. Although the minimum sample size required was 22 patients per group, 30 patients were enrolled per group for statistical requirements of normality.

Patients who met the inclusion criteria were admitted to the orthopedic ward. The aim and methodology of the study were explained to all the patients, and informed written consent was obtained from all the patients. Patients were split into two groups depending on the surgical procedure undertaken: PFN was performed in group A, and PFP was performed in group B.

Implant allocation was the surgeon's preference-based and non-randomized and was subject to implant availability. Spinal anaesthesia was used for all procedures, and they were carried out by an orthopaedic consultant with over five years' post-fellowship experience and a trainee. Postoperative care and rehabilitation were according to institutional protocol. All patients were administered a first-generation cephalosporin one hour prior to and for 24 hours following the procedure according to institutional protocol.

Fracture classification: The AO/OTA system (31-A1 to A3) was preoperatively applied to classify all the fractures on anteroposterior and lateral X-rays. Where applicable, CT scans were also performed. The fractures were then designated as stable or unstable according to established criteria. The classifications were separately done by two consultants, and discrepancies were settled on a consensus basis.

Outcome definitions: The major outcome was time to fracture healing, radiologically assessed by bridging callus over at least three of four cortices on orthogonal films, and the ability to walk without pain. The orthogonal films were assessed by a blinded assessor. Primary outcomes were surgical time, SSI within 90 days, and length of stay in the hospital. Functional grading, such as the Harris Hip Score (HHS), has not been utilized in the present study and is noted as a disadvantage.

The follow-up of the study patients was carried out postoperatively at one, three, and four months in the outpatient department. The status of fracture union and wound healing was documented on each follow-up, along with clinical examination, routinely.

All the information was documented on a predesigned, standardized questionnaire and transferred to Statistical Product and Service Solutions (SPSS, version 23; IBM SPSS Statistics for Windows, Armonk, NY) for analysis. Quantitative information (e.g., age, duration of fracture, surgery time, length of hospital stay, and healing of the fracture) was expressed as mean ± SD or median with IQR based on the distribution. Qualitative information (e.g., gender, comorbidities, ASA class, and SSI) was expressed as frequencies and proportions.

The Shapiro-Wilk test was utilized for testing the normality of continuous variables. An independent samples t-test was utilized for normally distributed quantitative data to make between-group comparisons, and the chi-square or Fisher's exact test for categorical variables (e.g., SSI). Multivariable linear regression (union time and operative time) and logistic regression (SSI) were also performed, controlled for age, sex, ASA status, smoking, diabetes, fracture stability/AO subtype, time to operation, and surgeon. Confounder stratification was carried out for age, gender, residence (urban/rural), smoking status, diabetes, hypertension, and ASA grade. t-tests and chi-square/Fisher's exact tests were employed for post-stratification comparisons as suitable. A p-value ≤ 0.05 was utilized in marking statistical significance throughout the analysis.

## Results

Among the 60 patients, 29 (48.3%) had stable fracture patterns (31-A1 and selected 31-A2), while 31 (51.7%) had unstable fractures (comminuted 31-A2 and 31-A3). In the PFN group, unstable patterns were more frequent (20/30; 66.7%) compared to the PFP group (12/30; 40.0%). Conversely, stable fractures were relatively more common in the PFP group (18/30; 60.0%) than in the PFN group (10/30; 33.3%). This is presented in Table 2.

**Table 2:** Stratification of fracture patterns by AO/OTA classification and stability AO/OTA: AO Foundation and Orthopaedic - Trauma Association; PFN: proximal femoral nailing; PFP: proximal femur plate

AO/OTA Classification	Stability Category	PFN Group (n=30)	PFP Group (n=30)	Total (n=60)
31-A1 (simple two-part)	Stable	6 (20.0%)	10 (33.3%)	16 (26.7%)
31-A2 (multifragmentary, less comminution)	Stable	5 (16.7%)	8 (26.7%)	13 (21.7%)
31-A2 (comminuted, lateral wall compromise)	Unstable	11 (36.7%)	5 (16.7%)	16 (26.7%)
31-A3 (reverse oblique/subtrochanteric)	Unstable	8 (26.7%)	7 (23.3%)	15 (25.0%)

The mean duration of surgery was significantly longer in the PFN group (79.10 ± 12.78 minutes) compared to the PFP group (64.13 ± 12.92 minutes) (p < 0.0001). In our study, this longer operative time for PFN may be caused by a higher proportion of cases that required open reduction due to irreducible fracture patterns, in contrast to the more commonly encountered reducible intertrochanteric fractures managed with closed reduction on a fracture table.

Similarly, the mean fracture healing time was longer in the PFN group (12.57 ± 1.79 weeks) than in the PFP group (10.60 ± 1.10 weeks) (p < 0.0001). Normally, an earlier union is expected in intramedullary nailing due to preservation of the fracture hematoma in closed reduction. In our series, the longer healing time in the PFN group may be explained by the greater number of cases requiring open reduction, which can disrupt the fracture hematoma and potentially delay union.

While considering the SSIs, two patients (6.7%) in the PFN group and one patient (3.3%) in the PFP group developed infections. However, this difference was not statistically significant (p = 0.500), indicating comparable safety profiles between the two interventions. This is presented in Table 3.

**Table 3:** Comparison of primary outcomes

Outcome Measure	PFN (Mean ± SD / %)	PFP (Mean ± SD / %)	P-Value
Duration of Surgery (min)	79.10 ± 12.78	64.13 ± 12.92	< 0.0001
Fracture Healing Time (weeks)	12.57 ± 1.79	10.60 ± 1.10	< 0.0001
Surgical Site Infection (%)	2 (6.7%)	1 (3.3%)	0.500

The mean duration of surgery was much longer for patients treated with PFN as compared to those who received PFP. This significant difference persisted across various stratified subgroups.

When stratified by age, patients aged 50-70 years had a mean surgical time of 78.46 ± 13.68 minutes in the PFN group versus 66.00 ± 12.89 minutes in the PFP group (p = 0.002). Patients older than 70 years demonstrated an even greater disparity, with mean times of 81.67 ± 8.68 minutes for PFN versus 52.00 ± 1.63 minutes for PFP (p < 0.0001). Gender-based analysis revealed significantly longer operative durations for PFN in both males and females. In males duration of operation was 77.78 ± 12.59 for PFN vs. 63.65 ± 12.24 minutes for PFP (p = 0.001), and in females, it was 83.43 ± 13.40 versus 65.10 ± 14.84 minutes, respectively (p = 0.020).

When stratified by place of residence, PFN procedures in urban patients lasted an average of 79.80 ± 11.87 minutes compared to 64.44 ± 13.11 minutes for PFP (p = 0.002), while rural patients demonstrated a similar trend (78.40 ± 14.01 vs. 63.79 ± 13.19 minutes; p = 0.008). Similarly, smoking status did not alter the overall pattern, with smokers undergoing PFN having longer surgeries (78.93 ± 13.88 minutes) than those treated with PFP (62.33 ± 12.11 minutes; p = 0.002), and non-smokers showing comparable differences (79.25 ± 12.19 vs 65.93 ± 13.87 minutes; p = 0.008).

Among diabetic patients, PFN procedures took 83.08 ± 12.56 minutes on average, which is significantly longer than the 65.36 ± 13.14 minutes recorded for PFP (p = 0.003). Non-diabetics also experienced prolonged operative time with PFN (76.06 ± 12.44 versus 63.42 ± 13.10 minutes; p = 0.006). Finally, hypertensive patients in the PFN group had a mean surgery duration of 82.71 ± 9.73 minutes vs. 61.54 ± 14.91 minutes in the PFP group (p < 0.0001), while normotensive patients showed a significant but smaller difference (75.94 ± 14.52 vs. 66.12 ± 11.24 minutes; p = 0.037).

These subgroup analyses were performed as part of stratification to control for potential confounding factors in the statistical analysis, rather than to imply that patient demographics directly influence operative duration. The observed variability in mean times among subgroups is more likely related to case complexity and the proportion of unreducible fracture patterns requiring open reduction rather than to the demographic factors themselves.

When stratified by fracture stability, operative time in stable fracture patterns was shorter in the PFN group compared to PFP (62.10 ± 10.95 vs. 76.45 ± 11.32 minutes; p = 0.001). Conversely, for unstable fractures, PFN procedures required significantly longer operative times than PFP (81.20 ± 13.15 vs. 65.05 ± 11.84 minutes; p < 0.0001). This is presented in Table 4.

**Table 4:** Stratified analysis: duration of surgery (78.46 ± 13.68 minutes) PFN: proximal femoral nailing; PFP: proximal femur plate

Stratification Variable	PFN (Mean ± SD)	PFP (Mean ± SD)	P-value
Overall	79.10 ± 12.78	64.13 ± 12.92	<0.0001
Age 50–70	78.46 ± 13.68	66.00 ± 12.89	0.002
Age >70	81.67 ± 8.68	52.00 ± 1.63	<0.0001
Male	77.78 ± 12.59	63.65 ± 12.24	0.001
Female	83.43 ± 13.40	65.10 ± 14.84	0.020
Urban	79.80 ± 11.87	64.44 ± 13.11	0.002
Rural	78.40 ± 14.01	63.79 ± 13.19	0.008
Smoker	78.93 ± 13.88	62.33 ± 12.11	0.002
Non-smoker	79.25 ± 12.19	65.93 ± 13.87	0.008
Diabetic	83.08 ± 12.56	65.36 ± 13.14	0.003
Non-diabetic	76.06 ± 12.44	63.42 ± 13.10	0.006
Hypertensive	82.71 ± 9.73	61.54 ± 14.91	<0.0001
Normotensive	75.94 ± 14.52	66.12 ± 11.24	0.037
Stable fractures	62.10 ± 10.95	76.45 ± 11.32	0.001
Unstable fractures	81.20 ± 13.15	65.05 ± 11.84	<0.0001

The mean time taken for fracture healing was longer in patients treated with PFN compared to PFP across most subgroups. Overall, the healing duration was 12.57 ± 1.79 weeks in the PFN group versus 10.60 ± 1.10 weeks in the PFP group (p < 0.0001). Patients presenting within six hours of injury had fracture healing times of 12.45 ± 1.90 weeks for PFN and 10.00 ± 0.96 weeks for PFP (p < 0.0001). Those presenting after six hours showed healing durations of 12.80 ± 1.61 and 11.13 ± 0.95 weeks, respectively (p = 0.003). It is important to note that the ‘presentation time’ refers to the interval from injury to arrival at our facility and not the time to surgery. Given the 50-80-year age range of our patients, all underwent standard preoperative anaesthetic assessment, and surgery was performed after necessary optimization rather than within six hours of injury. Among patients aged 50-70, PFN resulted in significantly longer healing (13.08 ± 1.31 vs. 10.69 ± 1.12 weeks; p < 0.0001), but in those over 70, the difference was not statistically significant (10.50 ± 2.07 vs. 10.00 ± 0.81 weeks; p = 0.663).

Males exhibited longer healing in the PFN group (12.78 ± 1.59) compared to PFP (10.45 ± 1.14; p < 0.0001), while the difference was not significant in females (11.86 ± 2.34 vs. 10.90 ± 0.99; p = 0.263). Urban and rural patients both showed significantly delayed healing in the PFN group (urban: 12.73 ± 1.98 vs. 10.69 ± 1.01; p = 0.001, and rural: 12.40 ± 1.63 vs. 10.50 ± 1.22; p = 0.002). Smokers treated with PFN had longer healing durations than those treated with PFP (11.93 ± 2.05 vs. 10.40 ± 1.24; p = 0.021), and the difference was even more pronounced among non-smokers (13.13 ± 1.36 vs. 10.80 ± 0.94; p < 0.0001).

Among diabetics, fracture healing took longer with PFN (12.23 ± 2.00) than with PFP (10.64 ± 1.28; p = 0.034), and similarly among non-diabetics (12.82 ± 1.62 vs. 10.58 ± 1.01; p < 0.0001). Hypertensive and normotensive patients also experienced significantly prolonged healing with PFN compared to PFP (12.50 ± 1.69 vs. 10.69 ± 1.03; p = 0.003 and 12.63 ± 1.92 vs. 10.53 ± 1.17; p = 0.001, respectively).

Radiological union occurred significantly earlier in patients treated with PFP compared to PFN across both stability groups. For stable fractures, the mean healing time was 10.20 ± 0.96 weeks with PFP versus 11.85 ± 1.42 weeks with PFN (p = 0.002). In unstable fractures, healing was again faster with PFP (11.05 ± 1.21 weeks) compared to PFN (13.12 ± 1.65 weeks; p < 0.0001). This is presented in Table 5.

**Table 5:** Fracture healing time PFN: proximal femoral nailing; PFP: proximal femur plate

Stratification Variable	PFN (Mean ± SD)	PFP (Mean ± SD)	P-value
Overall	12.57 ± 1.79	10.60 ± 1.10	<0.0001
Fracture Duration ≤ 6 hrs	12.45 ± 1.90	10.00 ± 0.96	<0.0001
Fracture Duration > 6 hrs	12.80 ± 1.61	11.13 ± 0.95	0.003
Age 50–70	13.08 ± 1.31	10.69 ± 1.12	<0.0001
Age >70	10.50 ± 2.07	10.00 ± 0.81	0.663
Male	12.78 ± 1.59	10.45 ± 1.14	<0.0001
Female	11.86 ± 2.34	10.90 ± 0.99	0.263
Urban	12.73 ± 1.98	10.69 ± 1.01	0.001
Rural	12.40 ± 1.63	10.50 ± 1.22	0.002
Smoker	11.93 ± 2.05	10.40 ± 1.24	0.021
Non-smoker	13.13 ± 1.36	10.80 ± 0.94	<0.0001
Diabetic	12.23 ± 2.00	10.64 ± 1.28	0.034
Non-diabetic	12.82 ± 1.62	10.58 ± 1.01	<0.0001
Hypertensive	12.50 ± 1.69	10.69 ± 1.03	0.003
Normotensive	12.63 ± 1.92	10.53 ± 1.17	0.001
Stable fractures	11.85 ± 1.42	10.20 ± 0.96	0.002
Unstable fractures	13.12 ± 1.65	11.05 ± 1.21	<0.0001

## Discussion

Treatment of femoral intertrochanteric fractures has come a long way in the past three to four decades [9,10]. Nonsurgical treatment is associated with complications such as pressure sores, pneumonia, urinary tract infection, and deep venous thrombosis, all of which add to morbidity and potentially mortality [11,12]. Therefore, the current practice is to treat it with surgery.

While various implants were previously employed for fixation, they are largely obsolete today [13]. The choice of treatment today largely depends on the fracture type and the patient’s overall health [14]. Trochanteric fractures, most frequently encountered in older patients, have high morbidity and mortality. Trochanteric fractures are injuries to the proximal femur, greater and lesser trochanters. As time has passed, treatment of these fractures has progressed towards operative strategies such as PFN and PFP, both of which normalize hip joint biomechanics and allow for early mobilisation.

In our cohort, all fractures were classified using the AO/OTA system (31-A1, A2, A3) and categorized into stable and unstable patterns. This stratification is important, as outcomes of fixation are strongly influenced by fracture stability. Our subgroup analyses depicted that, although PFP showed shorter operative time and faster union in many patients, these trends were basically limited to stable AO/OTA 31-A1 and selected 31-A2 fractures. PFN remained the most suitable implant for unstable patterns (31-A2 comminuted and 31-A3 reverse oblique), a finding consistent with established literature.

PFN: In this method, an intramedullary nail is inserted through a minimally invasive technique, providing a stable fixation by anchoring into the femoral head and shaft. It allows early weight-bearing and fewer complications that are associated with prolonged immobilisation.

PFP: Here, a plate and screws are both screwed into the lateral femur to stabilise the fracture. While it requires a slightly broader incision than PFN, it is adjustable and suitable for a range of fracture patterns and bone qualities. However, in modern practice, its role is usually limited to selected stable fracture configurations, particularly in centers where resource constraints or implant availability influence surgical choice.

Intertrochanteric femoral fractures, extracapsular between the trochanters, make up 45% of hip fractures [13]. The cancellous bone and weight-bearing trabeculae in the area reduce the chance of avascular necrosis and nonunion compared to intracapsular fractures [14].

The PFN group had patients between the ages of 50 and 80 (median: 60.0 years), and the PFP group had patients between the ages of 50 and 79 (median: 58.0 years). A similar study found that the average ages were 64.82 ± 4.12 years (PFN) and 62.37 ± 3.18 years (PFP) [4]. There were 23 men (76.7%) and seven women (23.3%) in the PFN group and 20 men (66.7%) and 10 women (33.3%) in the PFP group. Karim et al. found a different distribution: 36.17% males and 63.83% females (PFN) and 32.14% males and 67.86% females (PFP) [4].

Two people (6.7%) in the PFN group and one person (3.3%) in the PFP group had an infection at the surgery site (p = 0.500). The PFN group’s mean surgery duration was 79.10 ± 12.78 minutes, which was significantly longer than the PFP group’s (64.13 ± 12.92 minutes; p = 0.0001). In a similar vein, the PFN group’s mean fracture healing time was 12.57 ± 1.79 weeks longer than the PFP group’s (10.60 ± 1.10 weeks; p = 0.0001). These results are consistent with those of Karim et al., who found that fracture union times were 14.31 ± 3.12 weeks and 12.41 ± 0.38 weeks, respectively, and that mean surgery durations were 75.12 ± 6.13 minutes (PFN) and 60.18 ± 5.12 minutes (PFP) [4].

Epidemiological studies suggest an increasing incidence of proximal femoral fractures because of the greater life expectancy of the global population [15]. Intertrochanteric fractures constitute 90% of hip fractures in the elderly, with complication rates of 20-30% and a mortality rate of approximately 17% [16-18]. In elderly individuals, these fractures result from minor trauma, whereas high-energy trauma is typically responsible in younger patients [17]. Operative treatment is considered the optimal approach in most cases, and implants such as dynamic hip screws, angular blade plates, and cephalomedullary nails are considered effective [18,19].

Limitations

The study was non-randomized, with implant choice dependent on surgeon preference and availability, introducing potential selection bias. Although stratification by AO/OTA classification and multivariable adjustment were performed, residual confounding cannot be excluded. The limitations of this study include its single-center design, small sample size, and short follow-up period (four months), which limit the ability to generalize these findings, as well as assess long-term outcomes, such as implant failure, late nonunion, or reoperation rate. Furthermore, some subgroup analyses (smoker vs. non-smoker, urban vs. rural) are exploratory and hypothesis-generating, rather than confirmatory analyses. Another limitation is the absence of evaluation of functional variables, including the HHS. This also obviates assessment of longer-term recovery and patient-reported outcomes, which should be considered in future research.

## Conclusions

PFP was associated with shorter operative time and earlier radiological union compared to PFN. These advantages were primarily observed in stable AO/OTA 31-A1 and selected 31-A2 fractures, whereas PFN remains the standard of care for unstable AO/OTA 31-A2 comminuted and A3 patterns. Thus, PFP may still be considered in selected stable cases, particularly in resource-limited settings, while PFN continues to be the preferred option for unstable fractures
